# A Rare Case of Neuroglial Heterotopia of Nasopharynx

**DOI:** 10.22038/ijorl.2025.88070.3960

**Published:** 2026

**Authors:** Yellur Kavitha, Upendra Kumar Joish

**Affiliations:** 1 *Professor, Department of ENT, SDM College of Medical Sciences and Hospital, Shri Dharmasthala Manjunatheshwara University, Dharwad, Karnataka, India.*; 2 *Professor, Department of Radiodiagnosis, SDM College of Medical Sciences and Hospital, Shri Dharmasthala Manjunatheshwara University, Dharwad, Karnataka, India.*

## Abstract

**Introduction:**

Neuroglial heterotopia (NGH), is a displaced mass of mature central neuroepithelial tissue unconnected to the brain proper. NGH presents mostly with respiratory distress, neck mass and feeding difficulties at early age.

**Case Report:**

In our case, the nasopharyngeal NGH, a nine years old female child presented with otalgia and reduced hearing of left ear. Otological examination showed secretory otitis media. On nasal endoscopy solitary, smooth, mucosa covered mass on the eustachian tube opening of left side blocking eustachian tube orifice. Imaging showed a well-defined, non-enhancing, isodense soft tissue lesion arising from left lateral wall of nasopharynx with complete opacification of middle ear and mastoid. Patient underwent left myringotomy and grommet insertion. Nasopharyngeal mass removed trans-nasally under endoscopic guidance. Histopathological examination of the mass showed features of Neuroglial heterotopia. Patient improved symptomatically.

**Conclusion:**

Possibility of NGH should be considered while evaluating children with congenital nasopharyngeal mass. Thorough clinical evaluation, radiological imaging to rule out intracranial connection and complete excision are necessary for successful management of such cases.

## Introduction

Neuroglial heterotopia (NGH) of head and neck, is a rare congenital developmental anomaly characterised by presence of mature central neuroepithelial tissue in the extracranial sites. As these lesions are commonly found in nose and nasopharyngeal areas, they are also termed as nasal glioma. 

NGH contain differentiated neuroectodermal derivates such as neurons, astrocytes, ependymal cells and choroid plexus. Neuroglial heterotopia is reported in about 5% of cases with congenital nasal masses. NGH present early in life either as nasal mass with airway compromise, feeding difficulties or as mass neck. 

Clinical presentation of nasopharyngeal NGH is usually with respiratory distress, at early age itself^1^. In our case, the nasopharyngeal NGH, the patient presented late and the symptoms at the time of presentation was mainly otological. 

## Case Report

A nine years old female child presented to our otorhinolaryngology outpatient department with intermittent left ear pain of 5 to 6 years duration associated with reduced hearing in left ear. Child has been seen at various hospitals and given symptomatic treatment but without much improvement. There was no history of prior trauma to ear or ear discharge. Child did not give history of any nasal or throat complaints. 

On examination of the left ear, the external ear was normal. Tympanic membrane was lustreless and grossly retracted (Sade’s Grade IV) with few tympanosclerotic patches. Right ear was normal. Tuning fork tests showed features of moderate conductive deafness of left ear which was confirmed by pure tone audiometry. Tympanogram of Type B on left ear (suggestive of middle ear effusion) and Type A on right ear was noted. Examination of external nose, anterior rhinoscopy was normal and throat had no abnormal findings. 

**Fig 1:**
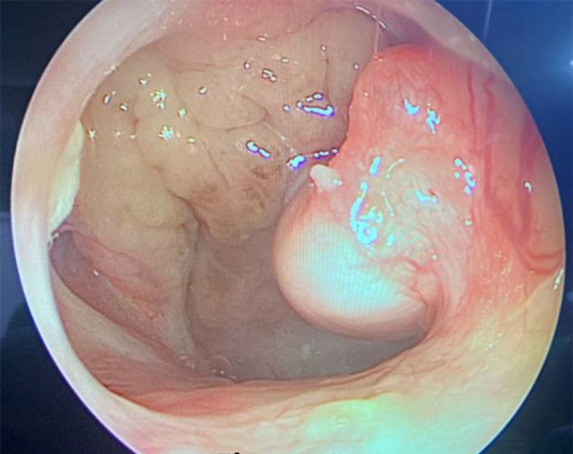
Nasal endoscopic view showing solitary, smooth, mucosa covered mass on the eustachian tube opening of left side obscuring tubal opening

As the child being in her middle childhood, was subjected to diagnostic nasal endoscopy to look for adenoid hypertrophy as it can cause eustachian tube obstruction and further lead to middle ear effusion. On nasal endoscopy a solitary, about 1.5 x1.5cm, smooth, mucosa covered, non-pulsatile mass with well-defined margins was seen on the eustachian tube opening of left side, blocking its orifice (Figure 1). The mass was sessile, firm in consistency and did not bleed on probing. The mass was limited to left half of nasopharyngeal cavity, not extending to oropharynx or nasal cavity. Roof of nasopharynx appeared free of lesion. Rest of the nasal cavity was normal. 

Due to its close proximity to cartilaginous end of eustachian tube, provisional diagnosis of chondroma was made. Hence the child was subjected to plain and contrast enhanced 128 slice MDCT scan of head and neck, to characterise the lesion.

**Fig 2:**
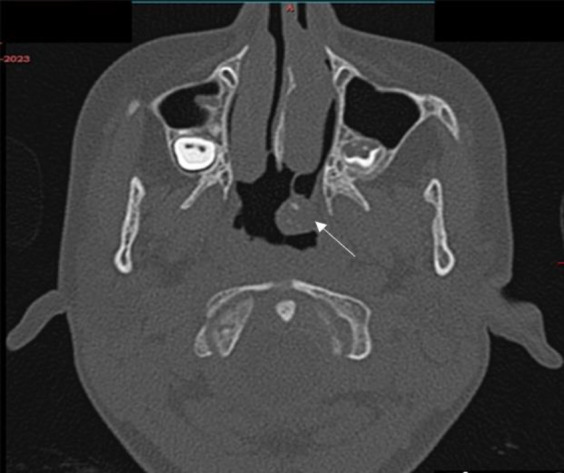
Well-defined non-enhancing isodense soft tissue lesion with hyperdense areas and calcific focus within noted arising from left lateral wall of nasopharynx of size 1.2x1.8x1.1cm (white arrow)

MDCT neck (plain and contrast) revealed a well-defined isodense soft tissue lesion with hyperdense areas and calcific focus within noted arising from left lateral wall of nasopharynx measuring 1.2 x 1.8 x 1.1 cm (AP x TR x CC) with no post-contrast enhancement (Figure 2). 

**Fig 3:**
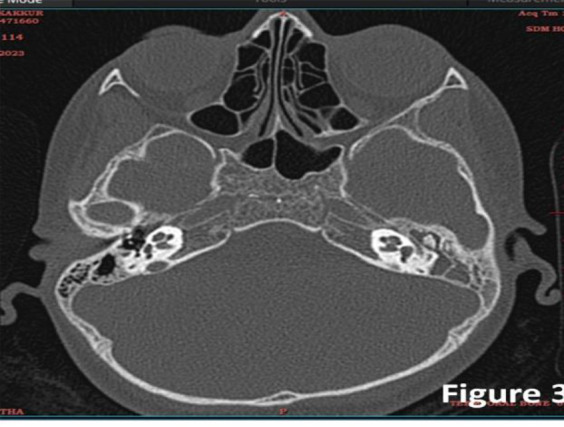
Complete opacification of left Eustachian tube, middle ear cavity, mastoid ad antrum, mastoid air cells. Sclerotic changes of petrous and mastoid parts of left mastoid air cells.

There was complete opacification of left eustachian tube, middle ear cavity, mastoid ad antrum and mastoid air cells with sclerotic changes of petrous and mastoid parts of left temporal bone (Figure 3). Skull base was found to be intact. Radiologically the lesion was diagnosed as adenoid mucous retention cyst of nasopharynx.

With features of middle ear effusion in a middle childhood patient having a well-defined cyst-like lesion on the corresponding side eustachian tube orifice, provisional diagnosis of adenoid mucus retention cyst was made. However, due to close proximity to cartilaginous end of eustachian tube and presence of hyperdense areas and calcific focus within the lesion as shown by CT scan imaging, possibility of chondroma was also considered.

**Fig 4:**
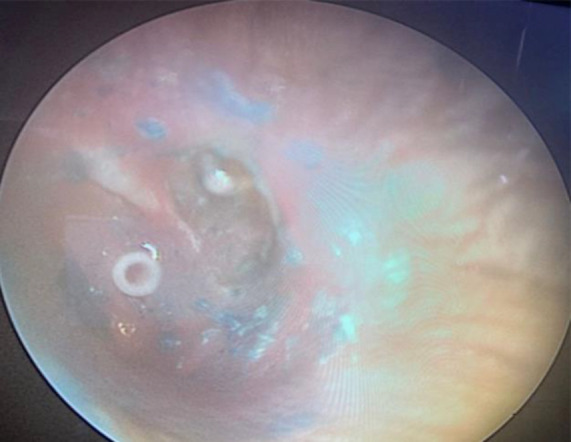
Myringotomy and Grommet placement

After obtaining informed written consent from parents, child was posted for surgery under general anesthesia. Myringotomy was done on left side, thick mucoid secretion from middle ear was drained. A grommet inserted (Figure 4).

**Fig 5:**
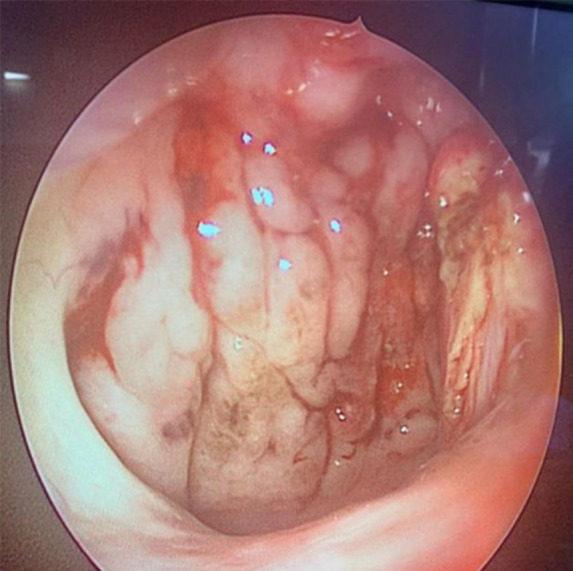
Intra operative image of nasopharyngeal mass excision

Under rigid nasal endoscopic guidance, nasopharyngeal mass was examined in detail which showed origin from nasopharyngeal end of left sided eustachian tube with rest of nasopharynx being free of lesion. Needle aspiration was attempted using a 22G spinal needle inserted into the lesion to look for any fluid within, however there was no fluid. The lesion was removed completely trans nasally using insulated monopolar diathermy with loop electrode without causing injury around eustachian tube orifice region (Figure 5). Post operative period was uneventful. Histopathological examination of lesion was reported as neuroglial heterotopia (Figure 6A, 6B, 6C). 

**Fig 6. A:**
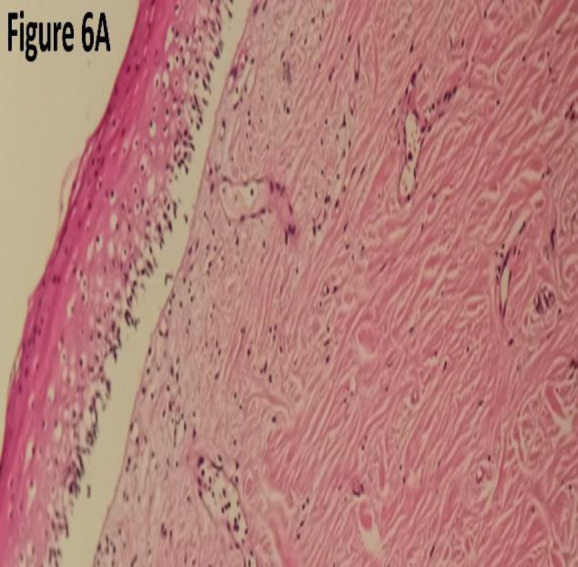
Polypoidal lesion covered with squamous epithelium. Sub epithelium showing dense collagen tissue

**Fig 6. B, C:**
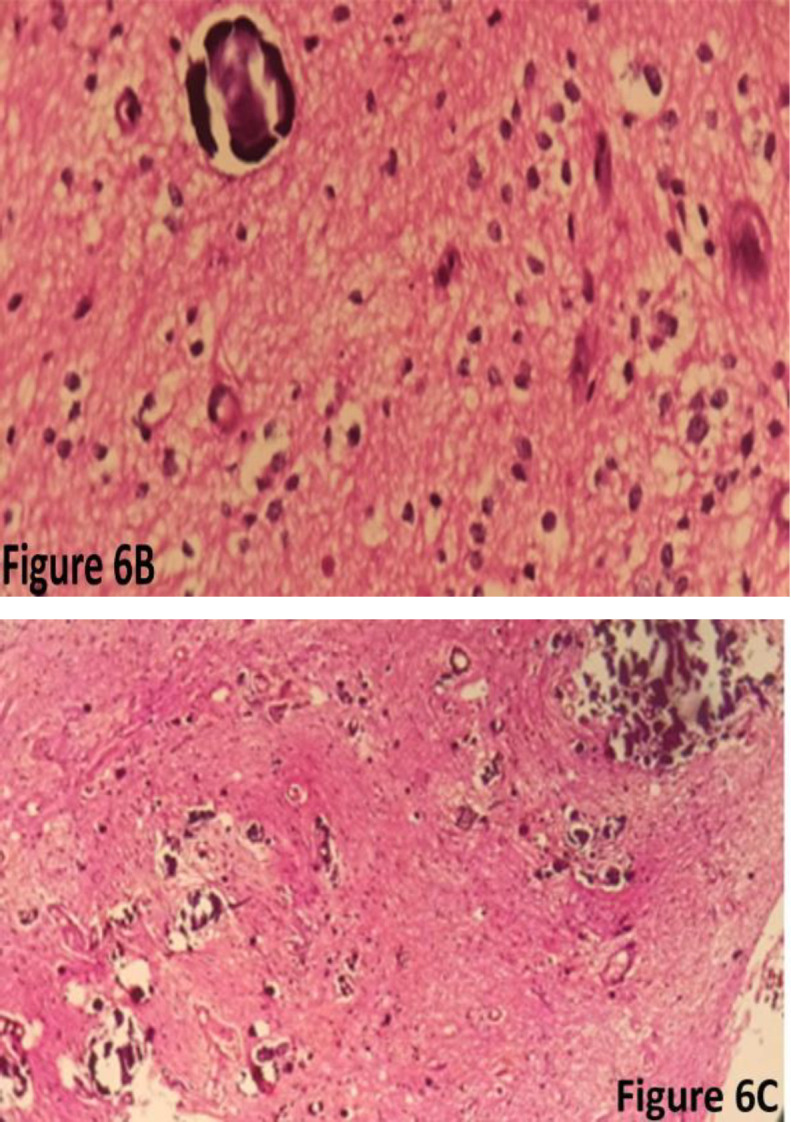
Mature brain tissue showing astrocytes, oligodendrocytes in a fibrillary background, Psammomatous calcification, hyalinised blood vessels

**Fig 7:**
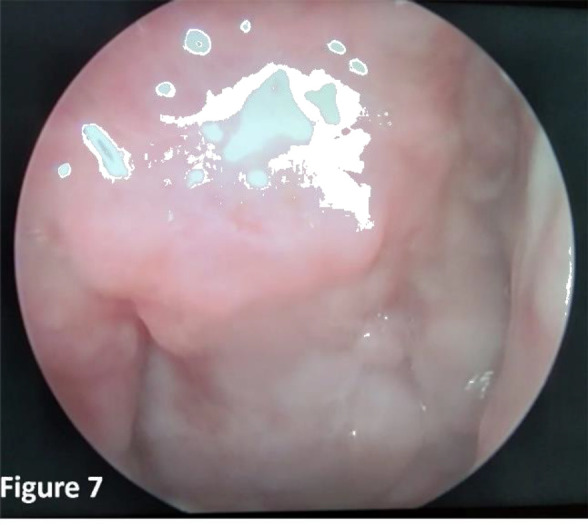
Follow up nasal endoscopy showing no evidence of recurrence

Patient is under follow up at regular intervals. Patient has shown improvement in audiological symptoms. Grommet is in situ and operative site in the nasopharynx has healed well (Figure 7). There is no evidence of recurrence of the lesion at the end of latest follow up (14 months post operative period).

## Discussion

Neuroglial heterotopia (NGH) arises as developmental malformation and mostly misdiagnosed as encephalocele, teratoma or haemangioma (2). NGH was first reported by Reid in 1852, however termed as NGH by Schmidt in 1900. Mostly they are solid lesions but sometime cystic due to production of fluid that resembles cerebrospinal fluid. By definition, NGH do not have continuity or communication with the cranial vault and its contents. Common site of origin of NGH is nasal cavity, rarely scalp, orbit, pterygopalatine fossa, pharynx, palate, lips, tongue, middle ear and neck (3). Underlying bone will be intact. Several theories of origin of NGH have been discussed in literature as its pathogenesis is not yet fully understood. One such theory states that NGH are nothing but encephaloceles that lost their cranial connection during development of the skull base (4). 

Another theory states that it arises from displaced totipotent neuroectodermal cells that developed into mature neural tissue (5). Currently, the encephalocele theory is most widely accepted. NGH can have variable histology, most consist being solid glial nests embedded in fibrous tissue and only 10% of lesions contain neurons. Astrocytes predominate and are often the only neuroepithelial element in nasal origin NGH. In pharyngeal lesions, histology is more complex, often containing ependyma-lined clefts and choroid plexus like formations. Clinical presentation of NGH can range from being asymptomatic and found incidentally to presenting with airway obstruction, feeding difficulty, mass effect or as a lump in the neck based on the site of origin. In literature, there are reports of NGH having undergone focal neoplastic transformation including an oligodendroglioma (6), an oligoastrocytoma and a melanotic neuroectodermal tumour of infancy (7,8). 

A probable instance of frontal lobe astrocytoma penetrating the cribriform plate to masquerade as a nasal glial heterotopia has also been described (9).  Table 1 shows various mode of presentations of nasopharyngeal NGH found in the recent literature. Imaging remains the mainstay in evaluation of NGH lesions. Both CT and MRI are complementary to each other. CT images are useful to study the relation of lesion with respect to skull base and also to look for bony defects or erosions. MRI is helpful to characterise the lesion either as solid or cystic, evidence of intercranial connections as well as differentiating tissue layers and spaces. 

Asymptomatic NGH does not warrant any treatment, unless its benign character is questionable or for aesthetic reasons. In symptomatic lesion, surgery is the treatment of choice after ruling out intracranial extension. Recurrence of NGH is rare if excised completely.

**Table 1:** Neuroglial heterotopia of nasopharynx described in literature

**Author**	**Year**	**Number of patients**	**Age and Gender**	**Imaging**	**Symptoms**
Karunakaran P^10^	2020	1	16 years, Female	MRI	Sore throat, feeling of a lump in throat
Baquero-Hoyos^11^	2021	1	9 months, Female	CT and MRI	Nasal obstruction, snoring, intermittent feeding difficulty, failure to thrive
Stucki B^12^	2025	2	12 months, female 21 days, Female	MRI CT	Cleft palate with palatal mass Pierre-Robins sequence, tongue-based airway obstruction
Gökler O^13^	2018	1	16 months, Male	MRI	Respiratory distress, Snoring

## Conclusion

NGH though rare, should be kept as differentials in evaluating children with congenital nasopharyngeal mass. Thorough clinical evaluation, radiological imaging and complete excision are the keys for successful management of such cases.
